# Reciprocal Field Transplant Experiment and Comparative Transcriptome Analysis Provide Insights Into Differences in Seed Germination Time of Two Populations From Different Geographic Regions of *Zostera marina* L.

**DOI:** 10.3389/fpls.2021.793060

**Published:** 2022-01-18

**Authors:** Yu Zhang, Shaochun Xu, Shidong Yue, Xiaomei Zhang, Yongliang Qiao, Mingjie Liu, Yi Zhou

**Affiliations:** ^1^CAS Key Laboratory of Marine Ecology and Environmental Sciences, Institute of Oceanology, Chinese Academy of Sciences, Qingdao, China; ^2^Laboratory for Marine Ecology and Environmental Science, Qingdao National Laboratory for Marine Science and Technology, Qingdao, China; ^3^Center for Ocean Mega-Science, Chinese Academy of Sciences, Qingdao, China; ^4^CAS Engineering Laboratory for Marine Ranching, Institute of Oceanology, Chinese Academy of Sciences, Qingdao, China; ^5^University of Chinese Academy of Sciences, Beijing, China; ^6^Shandong Province Key Laboratory of Experimental Marine Biology, Qingdao, China; ^7^Qingdao University of Science and Technology, Qingdao, China

**Keywords:** seagrass, biogeographical environment, seed germination, dormancy depth, transcriptome

## Abstract

Seagrasses are the only submerged marine higher plants, which can colonize the sea through sexual (*via* seeds) reproduction. The transition between seed dormancy and germination is an important ecological trait and a key stage in the life cycle of higher plants. According to our observations, the seeds of *Zostera marina* L. (eelgrass) in Swan Lake (SL) and Qingdao Bay (QB) in northern China have the same maturation time (summer) but different germination time. To investigate this phenomenon, we further carried out reciprocal transplantation experiment and transcriptome analysis. Results revealed that differences in the seed germination time between the two sites do exist and are determined by internal molecular mechanisms as opposed to environmental factors. Furthermore, we conducted comparative transcriptome analysis of seeds at the mature and early germination stages in both locations. The results that the number of genes related to energy, hormone and cell changes was higher in SL than in QB, could account for that the dormancy depth of seeds in SL was deeper than that in QB; consequently, the seeds in SL needed to mobilize more related genes to break dormancy and start germination. The results could have important practical implications for seagrass meadow restoration *via* seeds and provide in-depth and comprehensive data for understanding the molecular mechanisms related to seagrass seed germination.

## Introduction

Seagrass meadows are key ecosystems, and they are among the most threatened habitats on the planet. Seagrasses are the only submerged marine higher plants with a system of underground root and rhizome (Short et al., 2007). While colonizing the sedimentary shorelines of the world’s oceans, seagrasses have experienced genomic losses and gains to achieve the structural and physiological adaptations required for its marine lifestyle, arguably the most severe habitat shift ever accomplished by flowering plants (Olsen et al., 2016). As a functional group, they provide the basis for productive ecosystems along the coasts of all continents except Antarctica, and the ecosystem services they provide are on a par with tropical rainforests and coral reefs (Costanza et al., 1997; Fourqurean et al., 2012; Olsen et al., 2016). Seagrass species colonize the sea through both sexual (*via* seeds) and asexual (*via* clonal growth of rhizomes) reproduction (Phillips and Menez, 1988; Darnell et al., 2015; Zhang et al., 2020). Low pollination success, restricted dispersal of pollen and seeds, and low survival of seeds and seedlings, means that successful recruitment *via* sexual reproduction is restricted (Les, 1988; Laushman, 1993; Reusch, 2003); therefore, asexual reproduction is the main method of recruitment (Duarte and Sand-Jensen, 1990; Rasheed, 2004). Despite this, sexual reproduction is the only way to maintain the genetic diversity of the population, which improves resistance to adverse environments and resilience to disturbances (Ehlers et al., 2008; Cabaco and Santos, 2012; Xu et al., 2018). Furthermore, sexual reproduction also plays an important role in colonizing new habitats and recolonizing severely declined seagrass meadows by dispersing seeds from parental meadows (Harwell and Orth, 1999; Jarvis and Moore, 2010; Kendrick et al., 2012).

The seagrass *Zostera marina* L., or eelgrass, is the most widespread marine angiosperm species throughout the temperate northern hemisphere of the Pacific and Atlantic (Green et al., 2003; Olsen et al., 2016). Many studies have reported that sexual reproduction is crucial for the maintenance of within-species genetic diversity of eelgrass populations (Larkum et al., 2005; Kendrick et al., 2012; Ort et al., 2012). During sexual reproduction, eelgrass seeds are produced after flowering (Ort et al., 2012). No regional studies have directly documented long-distance dispersal, but genetic isolation by distance studies have suggested 150 km as an upper limit for the natural dispersal distance of *Z. marina* (Olsen et al., 2004; Muñiz-Salazar et al., 2005), which provides evidence for the colonization of new habitats with seeds. In most cases, once eelgrass seeds are released from the flowering shoots, they are deposited on or in the sediment, forming the seed bank (Fenner et al., 2005). A certain proportion of the seeds in the seed bank are maintained in a suitable physiological state conducive for germination to occur at an ecologically advantageous time. This reproductive strategy is vital for enhancing genetic diversity and avoiding the catastrophic loss of a plant population (Venable and Brown, 1988; Reynolds et al., 2013). Although much of the practical work on restoration of seagrass beds is carried out using adult seagrass plants, this approach has its limits. Apart from the huge labor and financial support required, the main problem is the potential loss of genetic diversity when adult plants are used to rebuild populations (Williams and Davis, 1996; Williams, 2001). In contrast, restoration of seagrass beds by seeding can avoid these problems, especially the loss of genetic diversity. Several restoration efforts have already shown that large areas of seagrass beds can be successfully restored by seeding (McGlathery et al., 2012; Orth et al., 2012, 2020; Orth and McGlathery, 2012), and the high genetic diversity in seagrasses restored using seeds rather than adult plants confers a greater level of ecosystem resilience to the restored seagrass meadows (Reynolds et al., 2012).

The transition between seed dormancy and germination is an important ecological trait and key stage in the life cycle of higher plants, which ensures species survival (Cadman et al., 2006; Holdsworth et al., 2008). Most seeds can survive long periods of unfavorable conditions and only germinate and grow into plants under the most favorable conditions. This ability stems, in part, from the existence of a dormancy mechanism (Cadman et al., 2006). Whether seeds are dormant or non-dormant and the type and depth of dormancy varies between species, or even between individuals of the same species (Baskin and Baskin, 2004). Seed dormancy and germination have been studied for decades in terrestrial higher plants (*Arabidopsis thaliana*, *Oryza sativa*, *Triticum aestivum*, *Hordeum vulgare*, etc.) at morphological, physiological, genetic, and molecular levels (Barrôco et al., 2005; Fait et al., 2006; Sreenivasulu et al., 2008; Howell et al., 2009; Galland et al., 2014; Han et al., 2017; He et al., 2020). As the only marine higher plant, the seed ecology of eelgrass has been studied to a certain extent (Churchill, 1992; Moore et al., 1993; Sugiura et al., 2009; Wang et al., 2016, 2017; Xu et al., 2016), but little is known about the molecular mechanisms of eelgrass seed germination.

We have observed that the seed germination time of *Z. marina* differed between Swan Lake (SL) and Qingdao Bay (QB) in northern China over a number of years. The time of seed maturation (summer time) is same in both places, but the time of germination is totally different. Seed germination in QB began in autumn of the same year as seed maturation, and most seeds germinated in October and November. In contrast, the seed germination period in SL is in spring of the year following seed maturation, from March to the end of May. Globally, a number of studies have found that the germination seasons of different geographical populations of eelgrass differ. For example, the seeds of eelgrass populations in the northeastern Pacific Ocean mainly germinate in spring and autumn (Kentula, 1982; Phillips and Backman, 1983), while populations in the northwestern Atlantic mainly germinate in autumn (Orth and Moore, 1986; Jarvis and Moore, 2010). Populations on European coasts mainly germinate in spring, but some can germinate in winter (Harrison, 1993; van Lent and Verschuure, 1994). Based on this phenomenon, we hypothesize that inherent genetic traits rather than environment determine the germination time of seeds, and the germination time does not change with the environment in which the seeds are planted. Therefore, we designed a field experiment in which seeds collected from QB and SL were reciprocally transplanted and their germination time was recorded. Comparative transcriptome analysis was then carried out to analyze the different events in eelgrass seed germination between SL and QB, which provides in-depth and comprehensive information for researchers relating to the molecular mechanism of seagrass seed germination.

## Materials and Methods

### Study Sites

**Figure 1:**
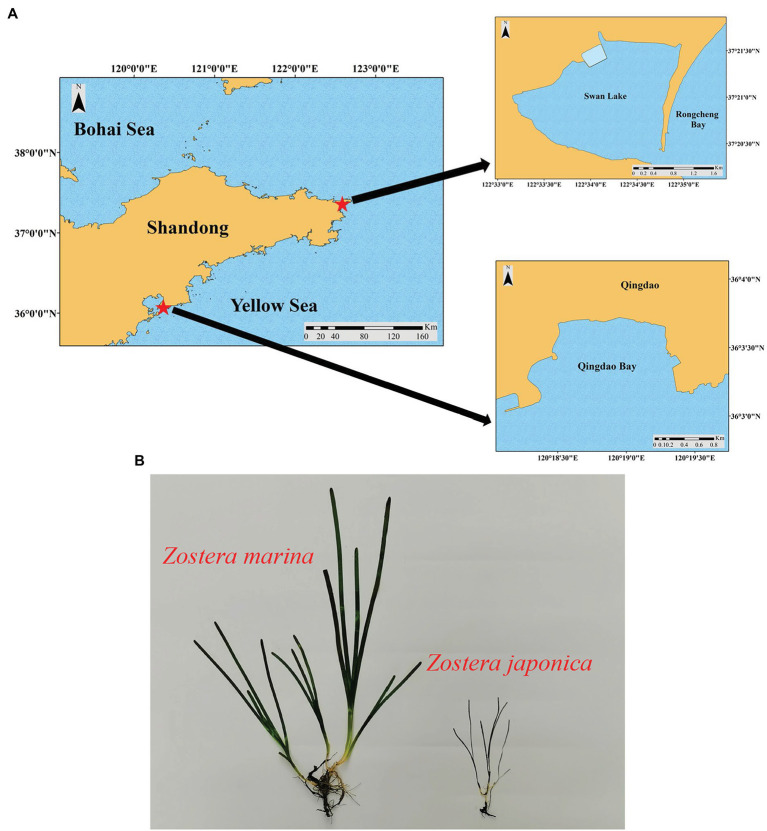
**(A)** The geographical location of Swan Lake (SL) and Qingdao Bay (QB). **(B)** Morphological comparison between *Zostera marina* and *Z. japonica* (QB, in winter).

SL (37°21′N, 122°34′E) is a marine lagoon in Weihai City, northern China covering an area of 4.8 km^2^ (Figure 1). SL is connected to the Yellow Sea by a narrow inlet that is 86 m wide (Zhang et al., 2020) and experiences irregular semidiurnal mixed tides, with a mean tidal range of 1.65 m. According to an investigation of environmental variables carried out by Xu et al. (2018), the water temperature shows an annual pattern ranging from −2.30 (February) to 25.60°C (August), and the annual average water temperature was 16.13 ± 7.76°C. Salinity ranges from 31.3 to 33.7 in SL. The sediment in the lake is mainly sandy and the daily photosynthetic photon flux densities increased from January and were highest in the summer months (June–July) (Xu et al., 2018). This lagoon is a suitable habitat for seagrasses with both *Zostera japonica* and *Z. marina* present (Zhou et al., 2015). *Zostera japonica* mainly occurs in the narrow mid-upper intertidal zone (Zhang et al., 2015, 2020); while *Z. marina* grows in the lower intertidal and subtidal zone (Zhou et al., 2015; Xu et al., 2018, 2019). There is a large difference in size between the two species, and the shoot height of *Z. japonica* and *Z. marina* in SL is shown in Table 1.

**Table 1:** Shoot heights of *Zostera marina* and *Zostera japonica* in QB and SL.

Sites	Species	Summer	Winter	References
QB	*Zostera marina*	80.23 ± 13.83 cm	20.98 ± 10.73 cm	Xu et al., 2018
	*Zostera japonica*	20.53 ± 3.66 cm	13.71 ± 1.70 cm	Zhang et al., 2020
SL	*Zostera marina*	75.60 ± 20.86 cm	18.65 ± 5.29 cm	Xu et al., 2018
	*Zostera japonica*	20.21 ± 4.59 cm	5.77 ± 0.19 cm	Zhang et al., 2020

QB (36°03′N, 120°20′E) is an open bay in Qingdao City, northern China (Figure 1A). The tides in QB are regular semidiurnal, with a mean tidal range of 4.8 m (Zhang et al., 2020). According to research carried out by Xu et al. (2018), the water temperature exhibits an annual pattern ranging from −0.50 (February) to 28.00°C (August), and the annual average water temperature is 17.94 ± 7.80°C. The salinity in QB ranges from 31.6 to 32.7. The sediment in the bay is mainly silt and sand, and the daily photosynthetic photon flux density is similar to that in SL (Xu et al., 2018). This bay has extensive areas of seagrass meadows dominated by *Z. marina*, but there also occurs a small area of *Z. japonica*. The distribution and shoot height (Table 1) of the two species in QB are similar to SL. The morphological comparison between *Z. japonica* and *Z. marina* is shown in Figure 1B.

### Experiment 1: Seed Transplant Experiment

#### Seed Collection

Reproductive shoots of *Z. marina* with spathes containing seeds were collected by hand from QB and SL in July 2017. Reproductive shoots were transported to the laboratory, where they were stored in a 600-μm mesh bag and placed in an aerated flow through tank (1 m × 1.2 m × 1.5 m). Mature seeds were naturally released from the flowering shoots and were then collected and sieved to remove any detritus and larger material. Following this, all seeds were kept in a running seawater tank until the initiation of seed reciprocal transplanting.

#### Seed Transplanting and Germination

Eelgrass seeds from SL were planted in QB on 21 August 2017. Seeds were placed in 12 polyvinyl chloride tubes (15 cm long, 1.2 cm inside diameter). Plastic mesh caps (1 mm × 1 mm) were placed at the bottom of each of the tubes, and the tubes were filled with dry sediment collected from the banks of QB. The sediment was saturated with natural seawater. Intact and full eelgrass seeds were selected, and groups of 50 seeds were planted in each tube at a burial depth of ~2–3 cm (Jarvis and Moore, 2015). Plastic mesh caps (5 mm × 5 mm) were placed at the top of each of the tubes to prevent sediment or seeds from washing out. Tubes were buried in the sediment, and the top of the tubes were level with the ground. At the same time, another 12 replicate tubes containing seeds from SL were also planted in SL for comparison. Meanwhile, 12 replicate tubes each containing 50 eelgrass seeds from QB were planted in SL on 25 August 2017. At the same time, another 12 replicate tubes containing seeds from QB were also planted in QB for comparison.

To examine the germination of seeds, three replicate tubes containing seeds from both QB and SL were sampled in October, November, December 2017, and April 2018 in both QB and SL. The tubes were sieved (0.7 mm) carefully *in situ* (Xu et al., 2018), and the retained materials were transported to the laboratory to count the number of germinated seeds. Germination of seeds was explicitly defined as not only the rupture of the seed coat but also the emergence and growth of the cotyledon (Churchill, 1983; Brenchley and Probert, 1998).

#### Data Analysis

All statistical analysis was carried out using R statistical software version 3.6.2 (R Core Team, 2016). Values were presented as means ± SE.

### Experiment 2: Transcriptomic Profiling Analysis

#### Seed Collection

Reproductive shoots of *Z. marina* with spathes containing seeds were collected by hand from QB and SL in July 2019. Collection and transportation of reproductive plants and harvesting of mature seeds was the same as described in section “Seed Collection in Experiment 1”. Molecular samples of mature seeds from the two sites were collected and named as QB-M and SL-M. The remaining seeds were kept in a circulating tank at room temperature in seawater. The status of seeds was observed once every 15 days until November, when the seeds collected from QB began to germinate in large numbers. By this time, molecular samples of seeds from QB (seeds in the germination stage but not yet germinated) and SL (seeds in the dormant stage and not germinated) were collected and were named as QB-G and SL-N, respectively. In March 2020, the seeds in the water tank collected from SL began to germinate in large numbers, and molecular samples of seeds from SL (seeds in the germination stage but not yet germinated) were collected at this time (SL-G). All molecular samples were immediately frozen in liquid nitrogen, and then stored in at −80°C. Four replicate samples, with each replicate containing approximately 10 seeds, were collected.

#### RNA Extraction, cDNA Library Construction, and Sequencing

Total RNA was extracted from *Z. marina* seed tissue using TRIzol® Reagent (Plant RNA Purification Reagent) according the manufacturer’s instructions and genomic DNA was removed using DNase I. The RNA was tested for concentration, purity, and integrity, and only high quality RNA was used for library building OD260/280 = 1.8–2.2, OD260/230 ≥ 2.0, RIN ≥ 6.5, 28S:18S ≥ 1.0, >1 μg. The RNA-sequencing (RNA-seq) transcriptome library was prepared using a TruSeqTM RNA sample preparation kit from Illumina (San Diego, CA) using 1 μg of total RNA. After quantification with TBS380, the paired-end RNA-seq sequencing library was sequenced with the Illumina HiSeq xten/NovaSeq 6000 sequencer (2 × 150 bp read length).

#### Quality Control, Read Mapping, and Sequence Annotation

The raw paired end reads were trimmed and quality control was carried out with SeqPrep and Sickle software to get high quality clean data. The genome of *Z. marina* was sequenced in 2016 (Olsen et al., 2016), and its annotation was updated in 2017. Therefore, *Z. marina* was used as the reference genome for subsequent sequence alignment and transcript assembly to get the mapped data. Based on the reference genome sequence, the mapped reads were assembled with Stringtie software and compared with the original genome annotation information. Finally, the functional annotations of the transcripts were summarized in the Non-Redundant Protein Sequence Database (NR), Swiss-Prot database (Swiss-Prot), the Protein families database (Pfam), Evolutionary genealogy of genes: Non-supervised Orthologous Groups (EggNOG), Gene Ontology (GO) database, and Kyoto Encyclopedia of Genes and Genomes (KEGG) database.

#### Expression Analysis and Enrichment Analysis

RSEM was used to quantify gene abundance, and the expression level of each transcript was calculated according to the transcripts per million reads method. Different expression analysis was performed using DESeq2 (1.24.0), and genes with p-adjust <0.05 and |log2FC| > 1 were considered to be significantly different expressed genes (DEGs). In addition, functional-enrichment analysis including GO and KEGG were performed to identify which DEGs were significantly enriched in terms of GO and metabolic pathways at Bonferroni-corrected *p* ≤ 0.05 compared with the whole-transcriptome background. GO functional enrichment and KEGG pathway enrichment analysis were carried out by Goatools^1^ and KOBAS.^2^

#### Quantitative PCR Verification

To evaluate the reliability of RNA-seq, we randomly selected eight genes (four each from SL and QB) for transcriptome validation using real-time quantitative polymerase chain reaction (RT-qPCR) and 18S rRNA was used as the internal reference gene. The RNA extracted as described in Section RNA Extraction was used as the template for reverse transcription to obtain cDNA. PCR results of the pre-experiment showed that the electrophoresis gel of each primer was a single bright band under specific conditions, indicating no specific amplification, and the primers could be used in subsequent experiments. The reaction system of RT-qPCR was as follows: 10 μl of 2× ChamQ SYBR Color qPCR Master MixPCR, 0.8 μl of both forward and reverse primers (5 uM each), 0.4 μl of 50× ROX Reference Dye, 2 μl of template (cDNA), and 6 μl of ddH_2_O, made up to a total volume of 20 μl. The cycle conditions of RT-qPCR were as follows: the initial step was 95°C for 5 min, and then 40 cycles (melting at 95°C for 5 s, annealing at 50°C for 30 s, and extension at 72°C for 40 s). The target gene and internal reference gene of each sample were subjected to RT-qPCR reaction. Each treatment group had three replicates, and each replicate sample had three multiple pores. The relative expression level was calculated using the 2^−ΔΔCt^ method. Data were analyzed by *t*-test, and differences were considered as statistically significant if *p* < 0.05.

## Results

### Seed Germination in the Reciprocal Seed Transplant Experiment

**Figure 2:**
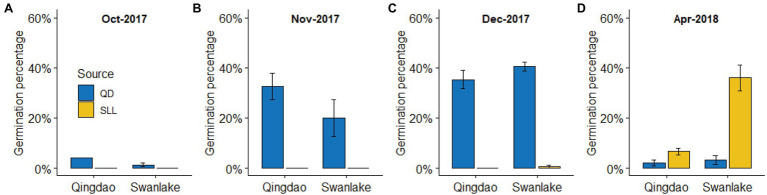
Germination percentages (means ± SE) of eelgrass seeds in a reciprocal transplant experiment at Qingdao Bay (QB) and Swan Lake (SL). **(A-D)** represent different observation time.

Seeds collected from QB had a higher seed germination rate in autumn (mainly November and December), regardless of whether they were planted in QB or SL. The highest germination rate occurred in SL in December 2017 (40.67% ± 1.76%); however, the germination rate was very low in spring (Figure 2). Seeds collected from SL had a very low seed germination rate in autumn, regardless of whether they were planted in QB or SL, with the germination rate close to 0; but the germination rate was relatively high in spring, with the germination rate 6.67 ± 1.33% and 36.00 ± 5.03% in QB and SL, respectively (Figure 2). Overall, seeds from QB mainly germinated during autumn, and seeds from SL mainly germinated during spring, which is consistent with our previous observations.

### Comparison of Seed Transcriptome in Autumn (QB-G vs. SL-N)

When a large number of seeds from QB began to germinate in the circulating water tank in the laboratory in autumn, un-germinated seeds from QB and SL were collected for transcriptome analysis. At this time, the seeds from QB should have been in the germination stage (QB-G), while the seeds from SL should have been in the non-germination or dormant stage (SL-N).

**Figure 3:**
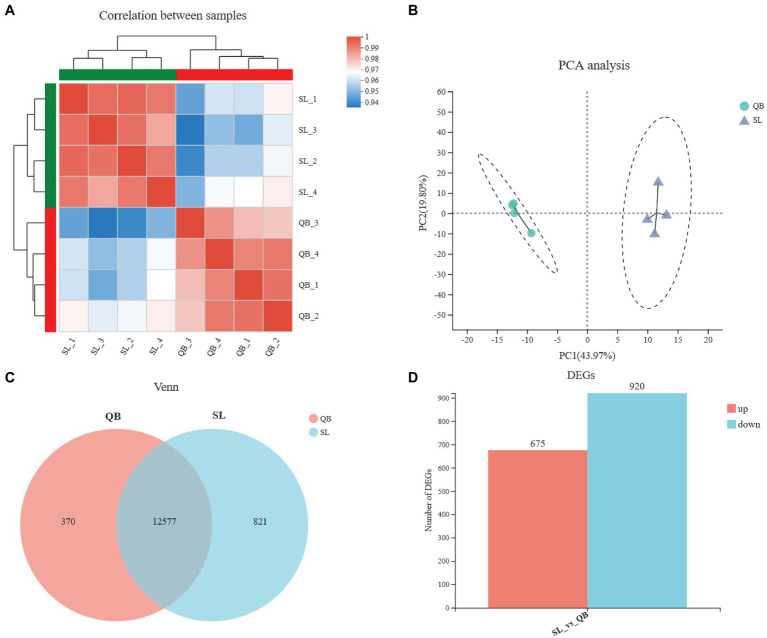
Statistical analysis of sequencing data of QB-G and SL-N. **(A)** Pearson correlation analysis. The right and bottom side are sample names, the left and top side are sample clusters, and different colors represent the degree of correlation between two samples. **(B)** Principal component analysis based on gene expression. The distance between samples represents the degree of similarity, the horizontal and vertical axes indicate the contribution of principal component 1 (PCA1) and principal component 2 (PCA2) to the differentiated samples, respectively. **(C)** Venn analysis. Numbers represent genes that are common or unique between groups. **(D)** Statistics of differentially expressed genes (DEGs). Red group represents upregulation, blue group represents downregulation, and the vertical axis represents the number of DEGs.

After transcriptome sequencing of eight samples from QB-G and SL-N were completed, a total of 54.36 GB of clean data were obtained, the clean data of all samples were above 5.89 GB, the percentage of Q30 base was above 93.27%, and the GC content of clean reads was about 44.31%. The clean reads of each sample were aligned with the designated reference genome, and the alignment rates ranged from 89.88 to 92.91%. The Pearson correlation coefficient between biological replicates was greater than 0.97 (Figure 3A), indicating a good repeatability and that the data are reliable. PCA analysis showed that there was a significant difference between the SL-N and QB-G samples (Figure 3B). Sequencing results showed that 12,577 genes were expressed in both SL-N and QB-G, and that 821 genes were uniquely expressed in SL-N, while 370 genes were uniquely expressed in QB-G, as shown in the Venn diagram (Figure 3C). A total of 1,595 DEGs were identified, including 675 upregulated genes and 920 downregulated genes (Figure 3D, the description of upregulation/downregulation was the result of gene changes in QB-G compared with SL-N).

**Figure 4:**
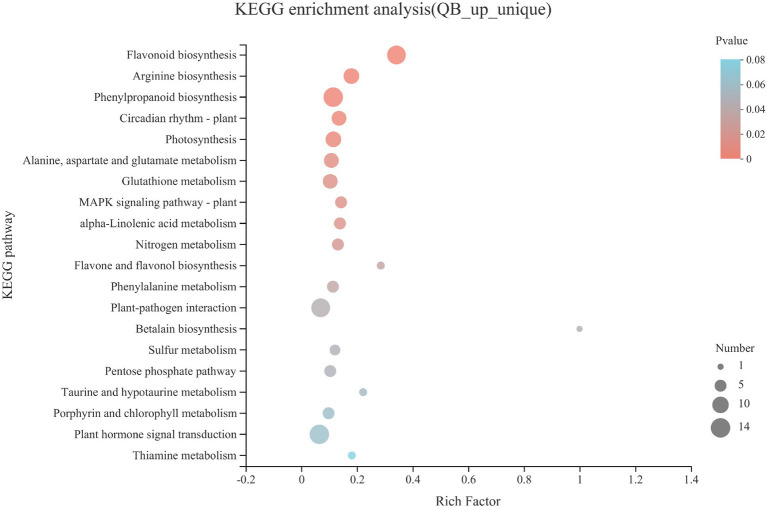
KEGG pathway enrichment analysis of gene sets including upregulated DEGs and uniquely expressed genes in QB-G. The ordinate represents the pathway name, and the abscissa is the rich factor; the larger the rich factor is, the greater the degree of enrichment. The size of the circle indicates the number of genes enriched in pathway, the circle color represents *value of p*.

The upregulated DEGs and uniquely expressed genes in QB-G were integrated into a gene set (QB_up_unique), and KEGG pathway enrichment analysis was performed (Figure 4). Flavonoid biosynthesis, arginine biosynthesis, phenylpropanoid biosynthesis, photosynthesis, alanine, aspartate and glutamate metabolism, glutathione metabolism, alpha-linolenic acid metabolism, nitrogen metabolism, flavone and flavonol biosynthesis, phenylalanine metabolism, and pentose phosphate pathways were significantly enriched (*p* < 0.05). On the whole, these significantly upregulated KEGG enrichment pathways are involved in energy metabolism, lipid metabolism, amino acid metabolism, and biosynthesis of secondary metabolites, which indicted that compared with the seeds from SL in the dormant stage, the seeds in the early stage of germination from QB had begun to initiate multiple metabolic pathways to mobilize the stored energy of seeds and gradually emerge from dormancy and start germination.

### Comparison of Transcriptome Between Early Germination Seeds and Mature Seeds at Two Sites

#### Overall Comparison of Total DEGs

**Figure 5:**
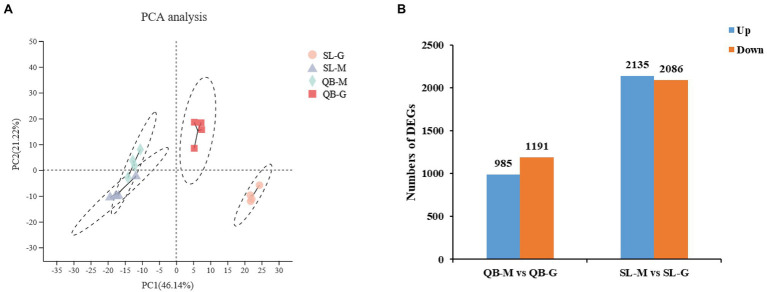
Statistical analysis of sequencing data of SL-M, SL-G, QB-M, and QB-G. **(A)** Principal component analysis based on gene expression. See Figure 3**B** for figure interpretation notes. **(B)** Statistics of differentially expressed genes (DEGs) between the seeds at the early germination stage and mature stage. The horizontal axis represents the comparison of seed germination at different sites, blue group represents upregulation, orange group represents downregulation, and the vertical axis represents the number of DEGs.

**Figure 6:**
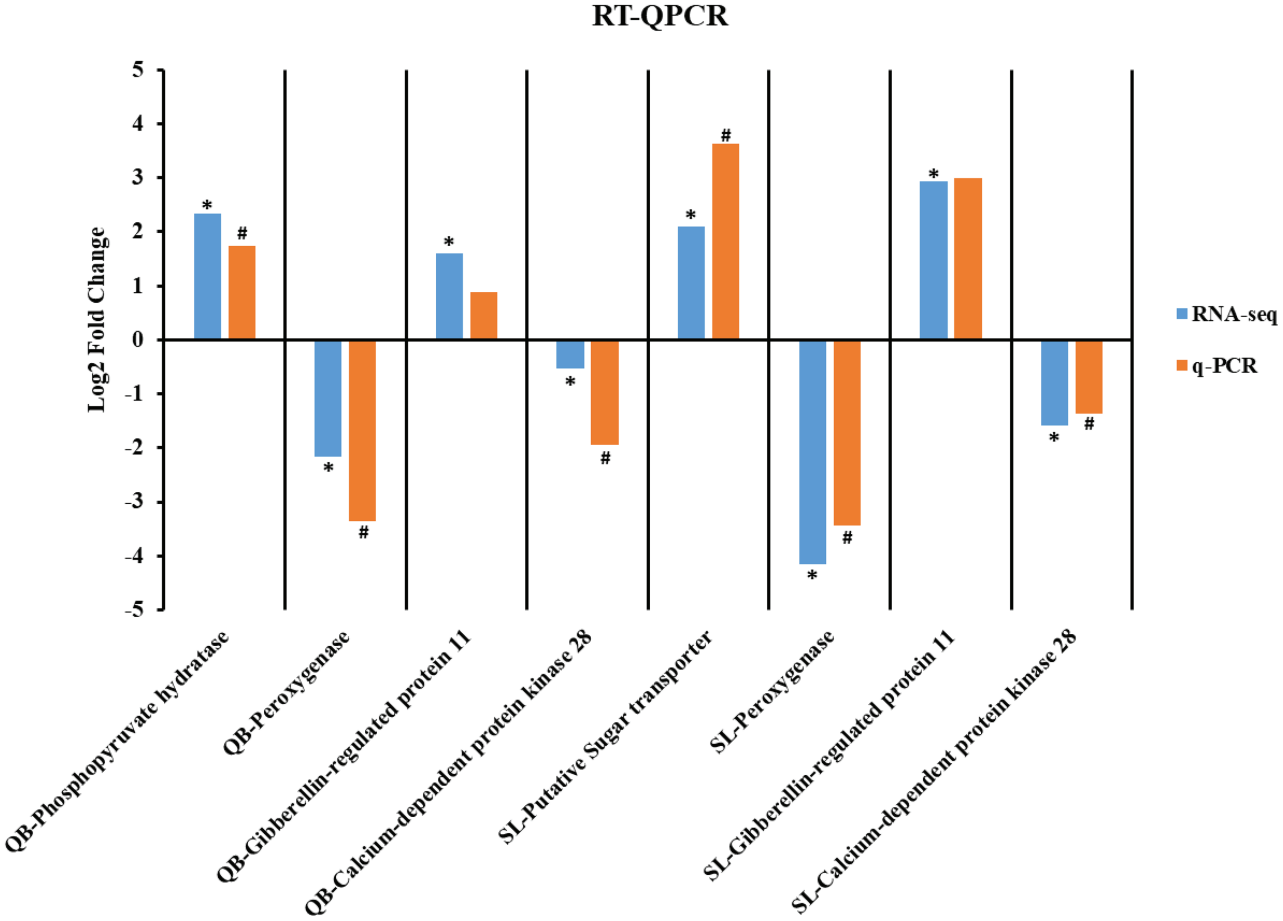
Comparison of gene expression trend based on RNA-seq and RT-qPCR. * and # indicate a significant difference in expression between seed in the mature stage and the germination stage in the RNA-seq and RT-qPCR, respectively.

Transcriptome sequencing of 16 samples from SL-M, SL-G, QB-M, and QB-G was completed. A total of 107.65 GB of clean data were obtained, clean data of all samples were above 5.88 GB, the percentage of Q30 base was above 93.32%, and the GC content of clean reads was about 44.40%. The clean reads of each sample were aligned with the designated reference genome, and the alignment rates ranged from 89.91 to 94.41%. Pearson correlation coefficient between biological replicates was greater than 0.97, indicating good repeatability and that the data are reliable. PCA analysis showed that four groups of samples were separated significantly in the PC1 × PC2 dimension (Figure 5A). In the first dimension (X-component) of the analysis, SL-M separated from SL-G, and QB-M separated from QB-G. In the second dimension (Y-component) of the analysis, SL-M grouped with QB-M, but QB-G separated from SL-G. The RT-qPCR results are shown in Figure 6, and the variation trend of the expression of these genes was consistent with the results of RNA-seq detection, indicating that the gene expression data obtained by RNA-seq was reliable.

**Figure 7:**
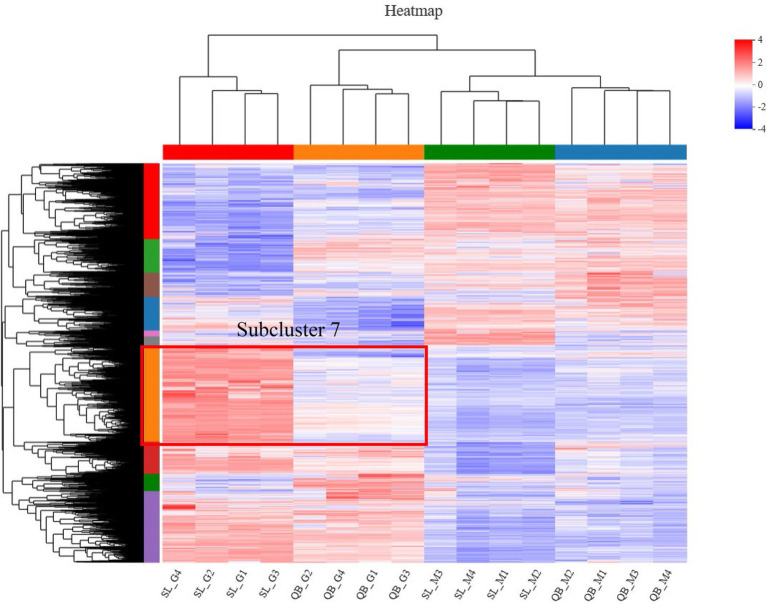
Cluster analysis of all-germination-genes set in two stages at two sites. Each column represents a sample and each row represents a gene. The color indicates the expression value of the gene after normalization in each sample. Red means the gene is highly expressed, blue means the gene is lowly expressed, and the number next to the color bar on the top right indicates the specific trend. The tree diagram of gene cluster and the module diagram of subcluster are shown on the left side, and the closer the two gene branches are, the closer their expression levels are. The upper part is the tree diagram of sample cluster, and the lower part is the name of the samples, and the closer the two sample branches are, the closer the expression patterns of all genes in these two samples are, that is, the closer the variation trends of gene expression levels are.

A total of 2,176 DEGs were identified by comparing QB-G and QB-M seeds, including 985 upregulated genes and 1,191 downregulated genes (Figure 5B). Meanwhile, a total of 4,221 DEGs were identified by comparing SL-G and SL-M seeds, including 2,135 upregulated genes and 2086 downregulated genes (Figure 5B). So, the number of DEGs in SL was nearly twice that in QB (the description of upregulated/downregulated was the result of gene changes in the germination stage compared with the mature stage). The DEGs identified in SL and QB were combined into a gene set, which was defined as all-germination-genes, and the cluster analysis of this gene set was performed simultaneously in two stages at two sites (Figure 7). Cluster analysis results showed that the gene expression patterns of SL-M and QB-M were similar, and the gene expression patterns of SL-G and QB-G were similar, but the expression patterns of SL-G and QB-G were significantly different in subcluster7.

**Figure 8:**
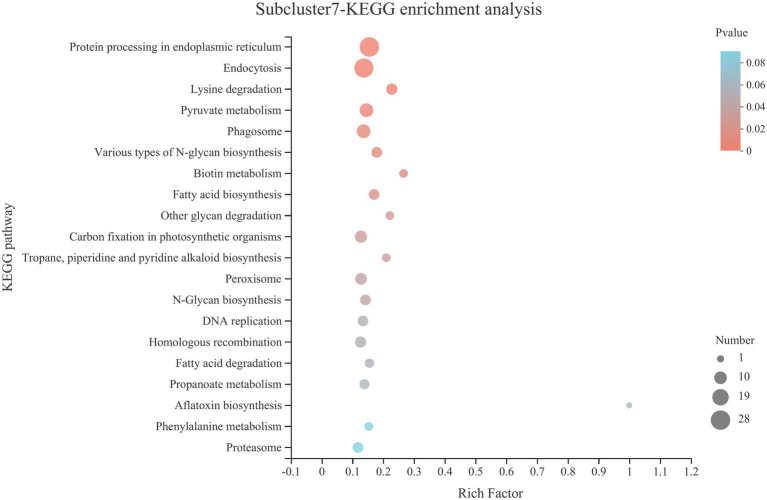
KEGG pathway enrichment analysis of genes in subcluster7. See Figure 4 for figure interpretation notes.

KEGG pathway statistics and enrichment analysis were further analyzed for genes in subcluster7 (Table 2 and Figure 8). The statistical results of the pathway are shown in Table 2, in which the processes, such as folding, sorting and degradation, transport and catabolism, carbohydrate metabolism and translation, contained the largest number of genes. KEGG pathway enrichment analysis showed that the process of protein processing in the endoplasmic reticulum was the most significant pathway (*p* < 0.0001).

**Table 2:** KEGG pathway statistics of genes in subcluster7.

First category	Second category	Gene number
Genetic information processing	Folding, sorting, and degradation	56
Cellular processes	Transport and catabolism	50
Metabolism	Carbohydrate metabolism	44
Genetic information processing	Translation	33
Environmental information processing	Signal transduction	28
Metabolism	Amino acid metabolism	24
Metabolism	Energy metabolism	23
Metabolism	Lipid metabolism	22
Genetic information processing	Replication and repair	20
Metabolism	Metabolism of cofactors and vitamins	18
Genetic information processing	Transcription	18
Metabolism	Glycan biosynthesis and metabolism	16
Organismal systems	Environmental adaptation	13
Metabolism	Metabolism of other amino acids	9
Metabolism	Biosynthesis of other secondary metabolites	8
Metabolism	Nucleotide metabolism	8
Metabolism	Metabolism of terpenoids and polyketides	6
Environmental information processing	Membrane transport	3

#### Pathways Associated With Energy Metabolism

**Figure 9:**
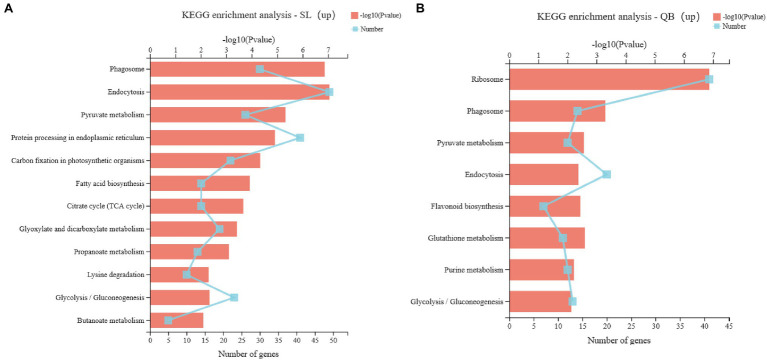
KEGG pathway enrichment of upregulated gene sets in Swan Lake (SL; **A**) and Qingdao Bay (QB; **B**). The vertical axis represents the name of KEGG Pathway; the lower abscissa represents the number of genes in this pathway, corresponding to different points on the broken line, the upper abscissa represents the significance level of enrichment, corresponding to the height of the column; the larger the value of – log10 (value of *p*) is, the more significantly enriched the KEGG pathway is.

Upregulated DEGs in SL were analyzed by KEGG pathway enrichment (Figure 9A). There were 12 significant enrichment pathways (*p* < 0.01), including seven pathways related to energy metabolism and carbohydrate metabolism: pyruvate metabolism, carbon fixation in photosynthetic organisms, citrate cycle, glyoxylate and dicarboxylate metabolism, propanoate metabolism, glycolysis/gluconeogenesis, and butanoate metabolism. Similarly, upregulated DEGs in QB were also analyzed by KEGG pathway enrichment (Figure 9B). There were eight significantly enrichment pathways (*p* < 0.01), but only two were related to carbohydrate metabolism, which were pyruvate metabolism and glycolysis/gluconeogenesis.

#### Comparison of ABA/GA Related Genes

**Figure 10:**
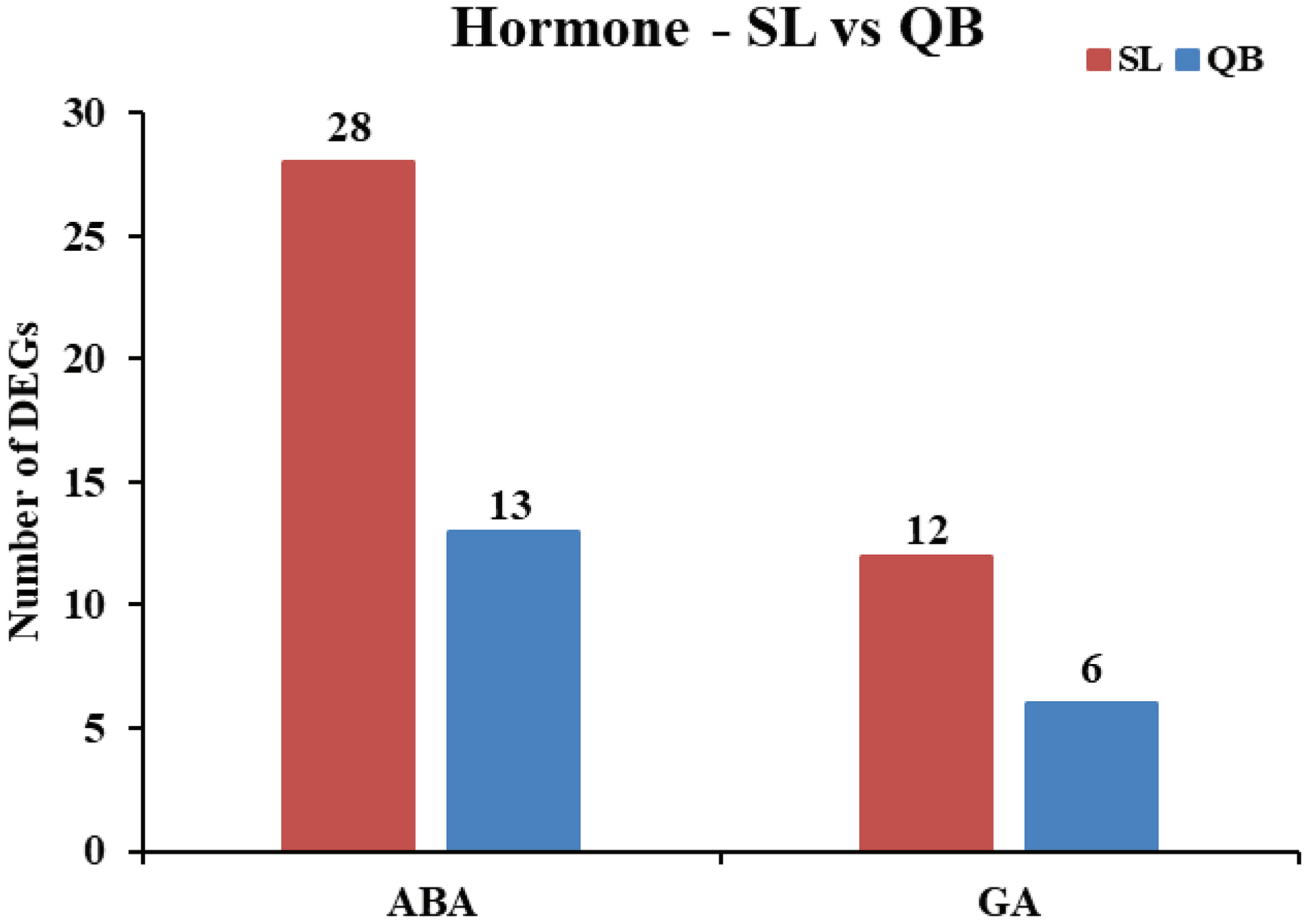
Statistics of DEGs related to negatively regulating abscisic acid (ABA) and positively regulating gibberellin (GA) in Swan Lake (SL) and Qingdao Bay (QB).

The plant hormone abscisic acid (ABA) is a positive regulator of dormancy induction and dormancy maintenance. A total of 38 genes related to ABA were found in the SL DEG set, including 17 upregulated DEGs and 21 downregulated DEGs (Figure 10). Further analysis revealed that nine of the 17 upregulated genes were related to ABA degradation, MYB transcription factor, and carbohydrate metabolism, and 19 of the 21 downregulated genes were related to the synthesis of ABA and response of ABA, which means that a total of 28 DEGs resulted in a decrease of ABA content. Similarly, a total of 21 genes related to ABA were found in the QB DEG set. Further analysis revealed that there were 13 DEGs resulting in the decrease of ABA content.

The plant hormone gibberellin (GA) is a positive regulator of dormancy breaking and germination induction. A total of 15 genes related to GA were found in the SL DEG set, including nine upregulated DEGs and six downregulated DEGs (Figure 10). Further analysis revealed that all the nine upregulated genes were related to the synthesis, perception, and regulation of GA, and three of the six downregulated genes were associated with the negative regulation of GA decomposition and synthesis, which means that a total of 12 DEGs resulted in the increase of GA content. Similarly, a total of seven genes related to GA were found in the QB DEG set. Further analysis revealed that there were six DEGs resulting in an increase of GA content.

#### Comparison of Genes Associated With Cell Changes

**Figure 11:**
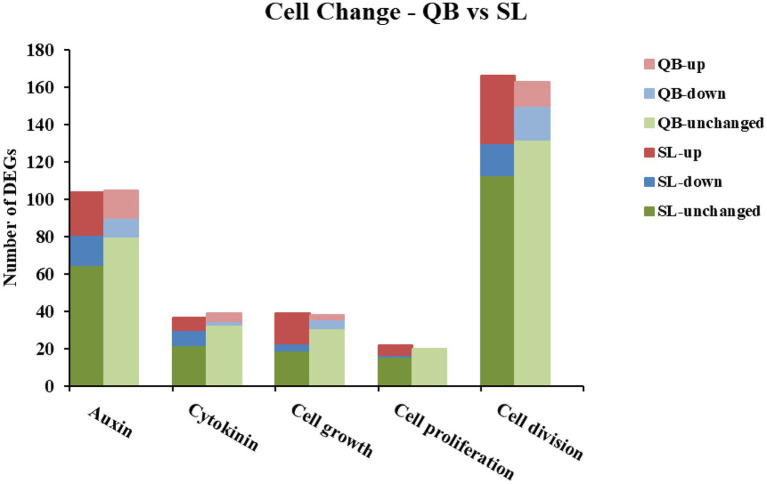
Statistics of genes associated with cell changes in Swan Lake (SL) and Qingdao Bay (QB).

Genes related to cell changes were identified in all genes from SL and QB (Figure 11), including genes related to cell growth, cell division, and cell proliferation. In addition, two plant hormones, auxin and cytokinin, were also included. The identification results were as follows: a total of 368 related genes were identified in SL, including 87 upregulated DEGs and 46 downregulated DEGs. A total of 365 related genes were identified in QB, including 34 upregulated DEGs and 35 downregulated DEGs. There were approximately 2.5 times more upregulated DEGs in SL than in QB. And, the number of downregulated DEGs in SL was slightly higher than that in QB.

## Discussion

According to the results of the seed germination time of the natural population of *Z. marina* observed for many years, the seeds from QB mainly germinate in autumn of the same year as seed maturation, and the seeds from SL mainly germinated in spring of the following year. In this study, we carried out a field experiment transplanting seeds from QB to SL and from SL to QB. The results showed that seeds from QB still germinated in autumn regardless of whether they were planted in QB or SL, and seeds from SL, whether planted in QB or SL, still germinated in the following spring. We also conducted comparative transcriptome analysis in autumn (germination stage of seeds in QB and dormant stage of seeds in SL). The results showed that genes involved in some metabolic pathways, such as energy metabolism, lipid metabolism, amino acid metabolism, and biosynthesis of secondary metabolites, were significantly upregulated in the seeds of QB compared with those of SL, which indicated that the seeds in QB were gradually emerging from dormancy and starting the germination process, while the seeds in SL were still dormant. Therefore, we believe that the factors that determine the seed germination time at the two sites are not environmental factors, but their different internal molecular mechanisms.

Comparative transcriptome analysis on seeds at the early germination stage in both places were conducted, mainly from four aspects: the overall comparison of DEGs in germination, the comparison of energy metabolism-related pathways, the comparison of major hormone-related genes controlling germination and the comparison of genes related to cell changes. Seed dormancy is a common phenomenon in the plant kingdom, which allows seeds to survive unfavorable conditions for a long time before germination and to establish plants under the most favorable conditions (Cadman et al., 2006). In many cases, seeds acquire primary dormancy during seed maturation and may enter a state of secondary dormancy if seeds are exposed to unsuitable temperature or lack adequate light (Finkelstein et al., 2008). Presumably, each seed is in a state somewhere along the continuum from deeply dormant to non-dormant, and the transition between different dormancy states is an active process involving changes in gene expression (Finkelstein et al., 2008). To investigate whether the dormancy states of the seeds in the two regions were the same, transcriptome analysis was performed to characterize gene expression in different physiological states. Principal component analysis was applied to the expression of all RNA-seq genes between two different physiological states at two sites. The analysis found that the first dimension grouped the mature seeds of the two sites together and separated them from the seeds of the germination stage of the two sites. The second dimension showed that the seeds in the same location had a certain similarity, and the seeds in different locations had certain differences, especially the seeds in the germination stage, SL and QB had great separation. Cluster analysis (heat map) also showed that the gene expression patterns of SL-G and QB-G were similar on the whole, but the number of upregulated genes of early germination seeds in SL was much higher than that in QB. In a review of molecular studies on seed dormancy, Finkelstein concluded that the expression level of dormancy related genes was to some extent correlated with dormancy depth (Finkelstein et al., 2008). Cadman, in his study of *Arabidopsis* seed germination, found that the number of genes involved in breaking dormancy or inducing germination was much higher at the post-ripening stage than at the dormant stage (Cadman et al., 2006). Based on this, we speculated that the dormancy depth of the seeds in SL was deeper than that in QB.

Embryos must mobilize carbon and energy sources to germinate and grow (Finkelstein et al., 2008). KEGG enrichment analysis of upregulated DEGs sets in early germination seeds from SL and QB revealed that pyruvate metabolism and glycolysis were both common and significantly enriched pathways, indicating that seeds in this period had started to mobilize reserves to prepare energy supply for the subsequent germination processes. Previous studies have shown that the photosynthesis and mineral uptake systems are not active during seed germination, and the energy for seed physiological activities is provided by reserve mobilization (Yu et al., 2014). In the early stages of germination, the energy generated by molecule degradation (e.g., glycolysis, glyoxylate cycle, and tricarboxylic acid cycle) is presumed to be a key determinant of germination vigor (Galland et al., 2014). Early activation of transcripts of starch and lipid reserve mobilization pathways can provide sucrose and hexose, which provide energy until the cotyledons become photoautotrophic plants (Sreenivasulu et al., 2008). Because of the limited penetration of oxygen into dense seed tissue, the large amount of energy required for seed development seems to depend primarily on glycolysis (Yang et al., 2007). Han et al. (2017) also hypothesized that glycolysis provides the embryo with a large amount of energy for the biosynthesis of new compounds and other metabolic requirements. In addition, our KEGG enrichment results also revealed that five other pathways related to energy metabolism were significantly enriched in seeds from SL, while only two pathways, pyruvate metabolism and glycolysis, were significantly enriched in seeds from QB. This demonstrated that the energy pathway mobilized in the early germination stage of *Z. marina* seeds in SL was more active than that in QB. We hypothesized that the amount of energy required to break dormancy to initiate germination was also different, because of the different depths of seed dormancy at different sites.

A large number of studies have shown that plant hormones are key regulators of seed dormancy and germination in terrestrial plants, including ABA, GA, ethylene, auxin, and brassinosteriods, with ABA and GA being the most important regulatory factors that play antagonistic roles in seed germination (Holdsworth et al., 2008; Nambara et al., 2010; Han and Yang, 2015). ABA is a positive regulator of induction and maintenance of dormancy, and a negative regulator of germination, while GA can counteract the effect of ABA, releasing dormancy and promoting seed germination (Kucera et al., 2005). ABA deficiency is associated with the loss of primary dormancy in mature seeds during seed development in many plants, and the overexpression of ABA biosynthetic genes can increase ABA content, enhance seed dormancy, or delay seed germination (Finkelstein et al., 2002; Nambara and Marion-Poll, 2003; Kushiro et al., 2004). Studies have shown that ABA restricts embryo growth potential by inhibiting water absorption (imbibition) and cell wall loosening, which are critical steps in the initiation of germination (Schopfer and Plachy, 1984; Gimeno-Gilles et al., 2009). GA, on the other hand, weakens the restriction of barrier tissues such as the endosperm or seed coat by inducing hydrolase, induces the mobilization of seed storage materials, and stimulates the expansion of the embryo to stimulate seed germination (Bewley, 1997). Studies have found that mutants with defects in the gene encoding GA biosynthetase in some species cannot germinate (Mitchum et al., 2006; Steber, 2008). In this study, it was found that the number of genes leading to the decrease of ABA content in SL during the early germination stage was higher than that in QB, with the number in SL being twice that in QB (SL:28 vs. QB:13). Among these DEGs, those related to ABA degradation, MYB transcription factors, and carbohydrate metabolism were significantly upregulated, while those related to ABA response or ABA signal transduction were significantly downregulated. Similarly, the number of genes responsible for the increase of GA content in SL was twice as high as that in QB (SL:12 vs. QB:6). In these DEGs, those related to the synthesis, perception, and regulation of GA were significantly upregulated, while those related to the decomposition of GA were significantly downregulated. As previously mentioned, ABA and GA are positive regulators of dormancy maintenance and dormancy breaking, respectively. Our results demonstrated that there were more genes related to emergence from dormancy when the seeds were about to germinate in SL seeds compared with those from QB. Therefore, it was speculated that the dormancy depth of the seeds of SL was deeper than QB.

Seed germination is a series of physiological and morphogenetic processes, where the plant embryo resumes growth after a period of quiescence (Barrôco et al., 2005). Considerable experimental evidence compels people to believe that the resumption of cell cycle activity is a specific feature of early germination (de Castro et al., 2000; Gallardo et al., 2002; Vázquez-Ramos and De la Paz Sánchez, 2003). In terrestrial dicotyledon seeds, the embryonic root first emerges from the seed, and then the embryonic shoot emerges from the seed after absorbing water. In terrestrial monocotyledon seeds, the cotyledon and radicle are covered by a coleoptile and coleorhiza, respectively, and the coleorhiza and radicle grow out of the seed in sequence. Following this, the coleoptile is pushed up until it reaches the surface (Nonogaki et al., 2010; Rajjou et al., 2012; Xue et al., 2021). However, eelgrass is a marine monocotyledon, whose seed germination process is slightly different to that of terrestrial seeds (Taylor, 1957). When the seeds germinate, the cotyledon sheath elongates first, then the first true leaf elongates from inside of the cotyledon sheath. Following this, the second true leaf and the adventitious roots emerge from inside of the cotyledon sheath (Sugiura et al., 2009). The radicle of eelgrass seeds never develops, so no primary root is formed and the adventitious root is the only true root of eelgrass seeds (Taylor, 1957). The rapid growth of the embryos, which eventually leads to the rupture of the covering layers and the emergence of the radicle, is considered to be the completion of seed germination in terrestrial plants (Barrôco et al., 2005). At this stage, the decision of individual embryo cells reenter the cell cycle or continue to be arrested is crucial for seedling formation. The establishment of plant shape and function depends on the ability of embryo cells to resume division and differentiation (Barrôco et al., 2005). In this study, we identified genes related to cell cycle and cell changes in SL and QB gene sets, which revealed that the number of upregulated DEGs in SL was 2.5 times that in QB at the early stage of germination. Therefore, we speculate that the dormancy depth of SL seeds is deeper, so more genes related to the cell cycle and cell changes need to be mobilized in the early stages of germination to initiate the subsequent germination process.

Germination is usually controlled by both internal factors, such as genetic control, and external factors, such as water temperature, which was very important in the field (Orth et al., 2000). According to Xu et al. (2018) and our observations over the years, although the latitude of SL is only 1°higher than that of QB, the temperature of SL is much lower than that of QB in winter. The intertidal zone of the QB have little chance to be covered by ice, while most of the intertidal zone in SL is covered by ice in winter, even the entire lagoon is covered by ice in some years. Phillips et al. (1983) reported that ice scouring in winter is an important source of eelgrass mortality at the northern margin of the range in the Bering Sea. Thus, eelgrass seeds from the SL population might be not acclimated to an autumn germination, because their seedlings cannot survive the severe winter. By contrast, seeds from the QB population can germinate in autumn and their seedlings can survive the relatively warm winter. Therefore, in autumn, seeds from SL would choose to go into a deeper level of dormancy to survive the cold winter, while seeds from QB would choose to germinate, which suggests that long-term differences in winter temperatures may be an important factor for the genetic differences.

## Conclusion

The results of field observations, the reciprocal transplant experiment, and molecular analysis were consistent, which supports our hypothesis that differences do exist in the seed germination period at two sites, and that these differences are not controlled by environmental factors but are determined by internal molecular mechanisms. Therefore, when using seed for seagrass bed restoration, it is necessary to take into account the germination characteristics of the donor seeds and choose the correct time for sowing. In addition, the comparative transcriptome analysis of seeds at the early germination stage at both sites from the aspects of overall germination DEGs, energy metabolism related pathways, major hormone genes controlling germination and genes related to cell changes, revealed that the dormancy depth of the seeds in SL was deeper than that in QB, and the long-term differences in winter temperature between the two sites might be the important factor for the genetic differences in seed germination time or dormancy depth between the two populations from different geographic regions.

## Data Availability Statement

All sequencing data are available through the NCBI Sequence Read Archive under the accession number PRJNA770030, and other data that support the findings of this study are available from the corresponding author, without undue reservation.

## Author Contributions

YZa: writing – original draft, conceptualization, methodology, and investigation. SX: writing – review and editing, conceptualization, and investigation (especially reciprocal seed transplant experiment). SY, XZ, YQ, and ML: investigation. YZo: funding acquisition, supervision, methodology, investigation, and writing – review and editing. All authors contributed to the article and approved the submitted version.

## Funding

This research was supported by the National Key R&D Program of China (2019YFD0901300), the National Science & Technology Basic Work Program (2015FY110600), the National Natural Science Foundation of China (No. 32000269), the Natural Science Foundation of Shandong Province (ZR2020QD106), the Key Research Project of Frontier Sciences of CAS (QYZDB-SSW-DQC041-1), and the Taishan Scholars Program (Distinguished Taishan Scholars).

## Conflict of Interest

The authors declare that the research was conducted in the absence of any commercial or financial relationships that could be construed as a potential conflict of interest.

## Publisher’s Note

All claims expressed in this article are solely those of the authors and do not necessarily represent those of their affiliated organizations, or those of the publisher, the editors and the reviewers. Any product that may be evaluated in this article, or claim that may be made by its manufacturer, is not guaranteed or endorsed by the publisher.

## Footnotes

^1^ https://github.com/tanghaibao/Goatools

^2^ http://kobas.cbi.pku.edu.cn/home.do
